# Counselling self-efficacy as a mediator between computer self-efficacy and attitudes toward tele-mental health among school counsellors in Malaysia

**DOI:** 10.1371/journal.pone.0335955

**Published:** 2025-11-06

**Authors:** Wei Rong Lee, Zaida Nor Zainudin, Engku Mardiah Engku Kamarudin

**Affiliations:** 1 Department of Counsellor Education and Counselling Psychology, Faculty of Educational Studies, Universiti Putra Malaysia, Serdang, Selangor, Malaysia; 2 Department of Psychology and Counselling, Faculty of Arts and Social Science, Universiti Tunku Abdul Rahman, Kampar, Perak, Malaysia; Regional Health Care and Social Agency of Lodi, ITALY

## Abstract

Tele-mental health has become an effective method to offer mental health services to a diverse and geographically dispersed population such as Malaysia. The success of tele-mental health programs relies heavily on the willingness and readiness of counsellors to embrace technology in their practice. This highlights the significance of counsellor attitudes towards technology in executing such initiatives. This study investigates how counselling self-efficacy mediates the relationship between computer self-efficacy and attitudes toward tele-mental health among Malaysian school counsellors. A cross-sectional study was conducted involving 348 school counsellors randomly selected from two states in Malaysia. The participants completed three instruments to evaluate their counselling self-efficacy, computer self-efficacy, and attitudes towards tele-mental health. Correlation results showed significant positive relationships among all three variables. Mediation analysis using PROCESS Macro (Model 4) demonstrated that counselling self-efficacy partially mediated the relationship between computer self-efficacy and attitudes toward tele-mental health (β = .030, 95% CI [.003,.067], t = 2.75, p < .01). The direct effect remained significant (β = .122, p < .01), confirming partial mediation. These findings suggest that improving school counsellors’ confidence in technological and counselling skills may potentially improve favorable attitudes towards tele-mental health adoption. This study contributes to the counselling profession in preparing school counselors for delivery tele-mental health and supporting the integration of technological training in counselling development programs.

## Introduction

The rapid development of the Internet has significantly accelerated daily life, profoundly impacting human interactions, particularly in communication. In Malaysia, as of January 2023, there were 33.03 million Internet users, representing 96.8 percent of the country’s total population [1]. Internet-based communication has emerged as a pivotal aspect of daily life, particularly among the younger demographic [2]. Statistics indicate that 99.8%, or approximately 24.80 million youths, engage in Internet communication through social media platforms [1].

The technological advancements coupled with the widespread adoption of the Internet and associated digital tools have precipitated a paradigm shift in counselling services, transitioning from traditional face-to-face counselling to tele-counselling [3]. Tele-mental health services are increasingly emerging as a viable alternative to in-person counselling sessions in the country [4], particularly amid pandemics such as the ongoing epidemic, where traditional counselling approaches are impractical. This shift has impacted all counsellors, with school counsellors particularly affected when schools are closed and online classes are the norm during such public health crises.

Tele-mental health is increasingly being utilised as a substitute for traditional counselling methods among school counsellors during pandemics. In the Malaysian context, school counsellors, known as “Guru Bimbingan dan Kaunseling” (GBK), are trained professionals responsible for providing counselling and guidance services to students within the school setting. The timing for implementing tele-mental health in schools is opportune due to the rising usage of the internet and mobile devices among adolescents in Malaysia. According to the Malaysian Communications and Multimedia Commission (MCMC), 83.2% of children and adolescents utilise the internet and mobile phones, spending an average of more than 6 hours daily on these devices [5].

Tele-mental health is not a novel concept in the counselling field. In the 1960s, computer software programs such as ELIZA and PLATO pioneered tele-mental health in Western nations such as the United States, where individuals interacted with a computer rather than with therapists [6,7]. Asynchronous modes of communication such as email were introduced following these software programs. Asynchronous communication involves delayed interaction [8]. With technological advancements, synchronous connections in tele-mental health have become feasible, enabling counsellors and clients to engage simultaneously from different locations [9]. Synchronous modalities include live chat, video calls, and instant messaging [3,10].

Tele-mental health has significantly reshaped the counselling landscape, particularly for practitioners such as school counsellors, who engage with younger clientele. This is attributed to the increasing involvement of the younger generation in online social interactions [11]. Consequently, school counsellors must adapt to this rapidly evolving social milieu to effectively engage with their target younger demographic [12,13].

According to [7], there lies a difference in satisfaction level among Malaysian students between tele-mental health and face-to-face counselling. The research findings indicate that Malaysian students express greater satisfaction with tele-mental health than face-to-face counselling. One contributing factor to this discrepancy in satisfaction is the issues of anonymity [14] and easy access [15] inherent in tele-mental health services. Numerous prior works documented various advantages associated with tele-mental health, which may impact students’ decisions to opt for tele-mental health over traditional face-to-face counselling modalities.

The effectiveness of tele-mental health is comparable to that of face-to-face counselling [13,16,17]. One minor difference in the benefits between tele-mental health and traditional counselling lies in the aspect of anonymity [16,18]. Clients can seek therapy in ways that improve anonymity, security, and privacy, reducing potential negative perceptions associated with seeking counselling [19]. Moreover, anonymity offers clients greater control, particularly for those uncomfortable with face-to-face counselling due to social anxiety [16,18].

The popularity and advantages of tele-mental health as an alternative counselling method have brought it closer to schools. However, recent research indicates that increasing the availability of tele-mental health services in schools may lead to increased workload for school counsellors [20]. The confidence to implement tele-mental health and the challenges faced by school counsellors in integrating tele-mental health into their daily tasks are discussed [10,21]. This challenge may result from school counsellors lacking proper training in tele-mental health and being unfamiliar with its techniques [11].

The ability to use technology has also been identified as a hurdle for school counsellors when incorporating tele-mental health into their routines [22]. According to [23], school counsellors with a high level of technological proficiency are more likely to embrace tele-mental health. Technological competence is a crucial aspect of readiness to adopt tele-mental health [22]. Professional development should focus on skills and the recent developments in adolescent online culture. School counsellors may come across difficulties related to adolescents who communicate online using language that is unfamiliar to them [10,21].

Therefore, this study aims to explore the relationship between counsellors’ attitudes toward tele-mental health and their self-efficacy in computer use, with counselling self-efficacy mediating this relationship. This study aims to establish a foundational understanding for future studies focusing on how self-efficacy influences attitudes toward tele-mental health. It also investigates whether counselling self-efficacy mediates the relationship between computer self-efficacy and attitude toward tele-mental health among school counsellors in Malaysia.

## Literature review

### Computer self-efficacy

According to [24], Computer self-efficacy is defined as an individual’s belief in their abilities to operate a computer. They propose that what has been accomplished previously is not as important as deciding what one might do next. [25] have revised the definition of computer self-efficacy, aligning it more closely with Bandura’s self-efficacy conceptualization [26]. Computer self-efficacy refers to individuals’ confidence in their abilities to plan and execute the necessary actions to successfully perform specific tasks in specific situations, such as tasks involving computer use [25,26]. It is considered the most critical determinant of computer-related ability and computer use.

In this study, computer self-efficacy is defined as an individual’s confidence in their ability to work with a desktop or laptop personal computer [27]. With advancements in computer technology, the definition of computer self-efficacy has evolved. The level of computer self-efficacy changes in response to technological advancements [27]. Perception of computer self-efficacy influences expectations regarding computer use. Individuals with low computer self-efficacy may feel inadequate in operating a computer, leading to reduced long-term computer utilisation. Conversely, high computer self-efficacy reduces computer-related anxieties and enhances employee performance and adoption of new technology [28].

### Counselling self-efficacy

Counsellors’ self-efficacy plays a vital role in their professional competence [29]. The therapist’s belief in themselves impacts the overall effectiveness of the counselling process [30]. Specifically, counselors’ self-efficacy is tied to their competency, which is called counselling self-efficacy. “Counselling self-efficacy” refers to an individual’s beliefs and expectations regarding their ability to guide a client successfully [31,32]. It encompasses counsellors’ confidence in performing counselling-related activities such as counselling sessions and development programmes, regardless of their educational background, years of experience, or counselling techniques [30].

Numerous prior studies on counselling self-efficacy have been conducted, focusing on various variables such as multicultural competency, counsellor performance, and training programmes [33]. [34] discovered a correlation between counselling skills and self-efficacy in counselling. The Skilled Counsellor Training Model (SCTM) has been shown to enhance counsellor self-efficacy and the effectiveness of counselling techniques [34]. [35] demonstrated that practising counsellors exhibit higher levels of self-efficacy in counselling after their practice. This is attributed to improving students’ knowledge and skills throughout the learning process.

### Attitude toward tele-mental health

Attitude towards behaviour refers to an individual’s inclination that regularly influences their thoughts, feelings, and actions regarding psychological objects [36]. Meanwhile, according to [37], attitude towards behaviour represents an individual’s perspective on their behaviour, which can manifest as positive or negative. [38] defines attitude towards conduct as the beliefs about the outcomes achievable when engaging in the associated activity and the importance attributed to these outcomes.

School counsellors have shown enthusiasm for integrating tele-mental health into their practice as an additional method of offering counselling to students [8,12]. However, the public’s perception of tele-mental health can be negatively impacted when there is insufficient training and professional development [39]. School counsellors have expressed concern about students’ lack of confidence in their technological knowledge and skills [21,23].

### Computer self-efficacy and attitude toward tele-mental health

Several studies have been conducted on computer self-efficacy and its implications. [40] investigated computer self-efficacy about various variables, including attitude toward computer use and behavioural intention. The findings indicate a clear relationship between perceived usefulness, attitude toward computer usage, computer self-efficacy, and the subsequent behavioural intention to utilise technology. This suggests that individuals with positive perceptions and high computer skills are more likely to use technology in their profession or work. However, this may deter them from seeking additional training and assistance [40].

Fang et al. [21] identified the advantages, obstacles, and user experiences associated with tele-mental health. They revealed that tele-mental health offers numerous advantages but also faces challenges, such as difficulties in internet connectivity. Consequently, individuals may feel uncomfortable using tele-mental health services if they lack confidence in technology. However, the findings suggest that training and practice can enhance individuals’ competence in using technology in tele-mental health settings [21].Top of Form.

Hassouneh and Alzoubi [41] conducted a study on the impact of modern technology on providing counselling services, with 125 counsellors participating. The findings revealed a lack of awareness of technology and the internet, a lack of interest in technology, and inefficiency were key reasons for counsellors’ lack of interest in using technology in their counselling sessions. Additionally, participants’ lack of trust in technology led to their inability to cope with technological advancements [41].

Golden [42] investigated the characteristics influencing school counsellors’ intention to use online counselling, with 85 school counsellors participating. The results suggest that school counsellors’ intentions to use online counselling services were influenced by their confidence in online counselling and their educational experience. This proves that the more self-assured school counsellors are, the more likely they are to use the Internet for counselling. School counsellors with greater experience in blended learning education, such as online and traditional learning experiences, are more inclined to adopt and use online counselling [42].

Abdillah et al. [43] conducted a study during the COVID-19 pandemic to assess the acceptance of school counsellors in using ICT for their counselling sessions. The research involved 214 school counsellors. The results indicated that perceived usefulness and computer self-efficacy were significant factors associated with the application of ICT. Counsellors who were confident in operating computers or the internet were more likely to use them in their counselling sessions [43].

### Counselling self-efficacy and attitude towards tele-mental health

Research focusing on e-counselling practitioners’ self-efficacy in counselling is relatively limited. Aliyev and Tunc [44] hypothesized that counsellors with a strong sense of self-efficacy are more effective at achieving desired goals during counselling sessions, especially in e-counselling settings where unforeseen scenarios may arise. The nature of e-counselling requires counsellors to be adaptable when utilizing various technology tools and addressing technical issues online. Strong self-efficacy among e-counsellors may indicate their capability to navigate e-counselling duties and activities despite potential obstacles not present in face-to-face environments.

Nagarajan and Yuvaraj [45] explored mental health professionals’ perceptions of using technology in counselling. The study involved 11 counsellors with 5–17 years of experience. Participants, all mental health professionals, expressed concerns about the lack of training for online counselling and the absence of technical and service-specific abilities. These concerns influenced their evaluation of whether to incorporate tele-mental health into their counselling sessions, as they lacked confidence in conducting tele-mental health without proper training.

Chen et al. [46] investigated the effects of clinical skills training on technology among counselling students. The study included 27 master’s level counselling students. The findings revealed that counselling students’ self-efficacy increased after online counselling skills training. This enhanced self-confidence in their ability to utilize online counselling approaches and related activities effectively. Students who received skilled counselling training exhibited a more positive attitude toward tele-mental health.

The potential of tele-mental health to broaden access to counselling services is recognized. However, counsellors have shown hesitancy in adopting this technology due to various factors. One primary factor is the lack of exposure to online tools during graduate training programmes, which may contribute to counsellors’ reluctance to embrace tele-mental health [47]. Despite the increasing prevalence of tele-mental health in mental health services, counsellor training has not kept pace with this demand. This has led to concerns about its efficacy [39,48]. Additionally, the shortage of licensed professional counsellors available to offer supervision for online counselling practice hampers the development of tele-mental health training programmes [3].

The deficiency in tele-mental health and computer training has raised concerns about counsellors’ effectiveness in providing tele-mental health services [10,49]. School counsellors also face similar challenges, lacking sufficient training in online tools and counselling skills, which discourages them from utilizing this new technology [10]. Self-efficacy plays a vital role in individuals’ ability to acquire counselling and computer skills, with varying levels of self-efficacy observed among individuals with different computer proficiency levels [50]. Research indicates that counsellors’ attitudes toward new technologies significantly impact their acceptance [51]. There was a lack of research about the relationship between computer self-efficacy and counselling self-efficacy. The connection between these two variables is still lacking exposure.

## Materials and methods

### Research design

The current study fills a gap in the literature review by examining the relationship between computer self-efficacy, counselling self-efficacy, and attitude towards tele-mental health. Most past studies have examined these variables individually, with very few exploring the mediating effect of counselling self-efficacy. This study tested this mediation model, in which a quantitative approach was applied. Quantitative methods are well-suited for testing hypotheses about the relationships between variables and measuring them numerically, wherein this approach allows statistical analysis to describe and explain what is observed [52]. The quantitative method can be utilized in many areas, including criminology, counselling, and research on tele-mental health as it evaluates and explains the statistical relationships. Cross-sectional research was used since participants only had to answer once during the study period. A multistage sampling technique was employed to collect the samples. In the first stage, Malaysia was divided into five geographical zones—Northern, Southern, Central, Eastern, and East Malaysia. The central zone was selected at random, marking the second stage. This zone consists of five states: Selangor, Negeri Sembilan, Melaka, Kuala Lumpur, and Putrajaya. In the final stage, a simple random sampling procedure was conducted, and two states—Selangor and Negeri Sembilan—were selected to ensure adequate coverage of the sample size. Data were analyzed using the Statistical Package for the Social Sciences (SPSS) version 27 with the Hayes PROCESS macro version 4.2. Model 4 was employed to conduct mediation analysis, with 5,000 bootstrap samples to generate bias-corrected 95% confidence intervals for the indirect effects. After a series of cleaning data, only data from participants who completed all measures were included in the final analysis.

### Ethical statements

Ethical approval was obtained from [anonymized]. Participant recruitment began on December 14, 2023, and concluded on December 14, 2024.

### Research instruments

Three instruments were utilized to collect data for this research. The research questionnaire consists of three instruments: the Computer Self-Efficacy Scale, the Counselling Self-Estimate Inventory (COSE), and the Online Counselling Attitude Survey (OCAS). An informed consent form was available on the first page, outlining the study objectives, potential risks and benefits, the voluntary nature of participation, privacy and confidentiality measures, and the contingency plan. The participants filled out this form when completing the questionnaire.

#### Computer self-efficacy scale (Howard, 2014).

The Computer Self-Efficacy Scale (CSES) is the first instrument utilized in this study. This scale evaluates an individual’s confidence in their ability to operate a desktop or laptop personal computer [27]. Comprising 12 different items, the instrument utilizes Likert scales for responses, ranging from 1 to 5. The selection of the Computer Self-Efficacy Scale [27] for this study was based on its status as the most recent version available for assessing computer self-efficacy and its simplicity. The total score, ranging from 12 to 60, reflects the level of computer self-efficacy and confidence, with higher scores indicating greater levels of proficiency. [27] reported a high level of internal consistency for the Computer Self-Efficacy Scale, with a Cronbach’s alpha of.95, indicating satisfactory and reliable consistency for each subscale.

#### Counselling Self-Estimate Inventory (COSE).

The Counselling Self-Estimate Inventory (COSE) developed by Larson et al. [31] serves as the second instrument in this study. This scale is utilized in counselling to assess counsellors’ beliefs or judgments regarding their abilities to advise a client effectively in the near future. The COSE comprises 37 items and is organized into five subdomains: micro skills, the counselling process, problematic client behaviours, cultural competency, and value awareness. These 5 subdomains comprise 18 positive items and 19 negative items.

Scores on the COSE can range from 37 to 222, with higher scores indicating greater confidence in one’s self-efficacy in counselling. A high score in any subdomain suggests that the counsellor possesses a high confidence level in their ability to provide effective counselling.

Larson et al. [31] reported good internal consistency for the COSE, with a Cronbach’s alpha of.93, indicating strong internal reliability. The subdomains demonstrated satisfactory internal consistency, with scores of.88 for micro skills,.87 for the counselling process,.80 for dealing with problematic client behaviours,.78 for cultural competence, and.62 for value awareness.

#### Online Counselling Attitude Survey (OCAS).

The final instrument utilized in this study is the Online Counselling Attitude Survey (OCAS) [53]. This tool assesses individuals’ attitudes toward online counselling, including evaluating disagreements among experts regarding its effectiveness and limitations. The OCAS comprises ten items, with five items measuring discomfort with online counselling (OC-D) and the other five measuring the value of online counselling (OC-V).

Responses on the OCAS were collected using Likert scales, ranging from 1 to 5, indicating varying degrees of agreement: strongly disagree, disagree, agree and disagree, agree, and strongly agree, respectively. The score was calculated by summing up the results of each item. Each subscale (OC-V and OC-D) ranged from 5 to 25 points, representing emotions, feelings, and reactions toward online counselling. Higher scores on the OC-V subscale indicate a positive attitude toward online counselling, whereas higher scores on the OC-D subscale indicate a negative attitude.

Rochlen et al. [53] reported test-retest reliability coefficients for the OC-V subscale as r = .88 and for the OC-D subscale as r = .77. They reported test-retest reliability coefficients for the OC-V as r = .93 and for the OC-D subscale as r = .91. The reliability coefficients of the OC-V and OC-D subscales for the OCAS were.84 and.78, respectively, indicating their reliability.

## Findings

### Mediation analysis

#### H1: Counselling self-efficacy mediates the relationship between computer self-efficacy and attitude toward tele-mental health.

The study examined the mediating effect of counselling self-efficacy on the relationship between computer self-efficacy and attitude toward tele-mental health. Mediation analysis was conducted using Hayes PROCESS macro (Model 4) with 5,000 bootstrap samples to assess the direct, indirect, and total effects.

**Fig 1:**
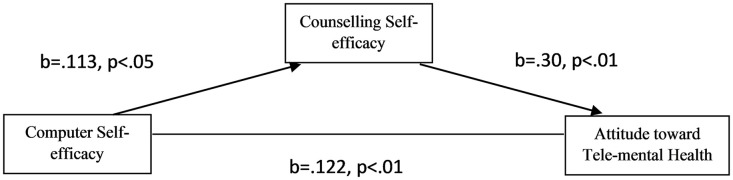
Mediation Model of Counselling Self-Efficacy Between Computer Self-Efficacy and Attitudes toward Tele-Mental Health.

Table 1 presents the mediation results. The results show that computer self-efficacy was significantly correlated with counselling self-efficacy (b = .113, p = .036). Counselling self-efficacy positively correlated with attitude toward tele-mental health (b = .265, p=<.001). The indirect effect of computer self-efficacy on attitude toward tele-mental health via counselling self-efficacy shows the result of significant (b = .030, 95% CI [.0026,.0619]). This indicates that the relationship between computer self-efficacy and attitude toward tele-mental health is affected by counselling self-efficacy. The direct effect of computer self-efficacy and attitude toward tele-mental health in the presence of a mediator, which is counselling self-efficacy, was significant (b = .122, p = .006). Therefore, counselling self-efficacy partially mediates the relationship between computer self-efficacy and attitude toward tele-mental health. The total effect also showed a significant relationship (b = .153, p = .001). The results indicated that school counselors with higher confidence in their computer abilities will have a favourable attitude toward tele-mental health, directly and indirectly through their confidence in counselling skills. Fig 1 summarizes the mediating model results.

**Table 1:** Mediation analysis summary.

Effect type	b	se	t	p	95% CI (LL,UL)
Total effect	0.1527	0.0465	3.29	.001	[.0613, .2441]
Direct effect	0.1227	0.0446	2.75	.006	[.0350, .2104]
Indirect effect	0.0300	0.0149	–	–	[.0026, .0619]
Path a	0.1133	0.0538	2.11	.036	[.0075, .2190]
Path b	0.2647	0.0443	5.98	<.001	[.1776, .3519]

Note. b = unstandardized regression coefficient, se = standard error, t = t-value, p = p-value, LL = lower limit; UL = upper limit of 95% CI.

Based on the results obtained, the relationship between the variables is significant. Table 2 shows the descriptive statistics and correlations among the study variables. The columns labelled 1,2, and 3 correspond to the variable: 1 equal to computer self-efficacy, 2 equal to counselling self-efficacy and 3 equal to attitudes toward tele-mental health. The correlation coefficient (r) indicates the strength and direction of the relationship between two variables. Therefore, a positive significant relationship between computer self-efficacy and attitude toward tele-mental health (r = .174, p < .01). This suggests that those with better computer skills will have a more positive attitude toward tele-mental health. The relationship between computer self-efficacy and counselling self-efficacy shows a positive, significant relationship (r = .113, p < .05), indicating that school counsellor who are more confident in their computer skills will also have greater counselling abilities. Likewise, counselling self-efficacy and attitude toward tele-mental health shows a positive correlation (r = .319, p=<.01), indicating that with higher counselling skills, school counsellor are more likely to view tele-mental health positively.

**Table 2:** Correlation analysis summary.

Variable	n	*M*	*SD*	1	2	3
1. Computer self-efficacy	348	3.50	.618	1	.113*	.174**
2. Counselling self-efficacy	348	4.10	.622	.113*	1	.319**
3. Attitude toward tele-mental health	348	3.20	.542	.174**	.319**	1

*. Correlation is significant at the 0.05 level (2 -tailed).

**. Correlation is significant at the 0.01 level (2 -tailed).

## Discussion

Our research has uncovered a significant relationship between counselling self-efficacy, computer self-efficacy, and attitudes toward tele-mental health among Malaysian school counsellor. The results indicated that counselling self-efficacy partially mediated this relationship, suggesting that computer self-efficacy is associated with attitudes toward tele-mental health both directly and indirectly through counsellors’ confidence in their counselling abilities. This implied that an increase in counselling self-efficacy is related to a higher level of computer self-efficacy and a more favourable attitude toward tele-mental health.

It is worth noting that previous studies have not explored the connection between computer self-efficacy and counselling self-efficacy in-depth. However, our findings are supported by the work of [43], which reported that school counsellors with higher confidence levels are more likely to use technology in their counselling practices. This is because a high level of computer self-efficacy increases the counsellor’s comfort level with conducting counselling sessions online. These results suggest that both computer and counselling self-efficacy may be important factors related to counsellors’ perceptions and practices.

According to [54], a counsellor’s years of experience and comfort with technology can predict their self-efficacy. This suggests that utilizing technology can improve a counsellor’s self-efficacy in online counselling. It enables them to overcome challenges in asynchronous communication and effectively address online counselling issues [11,54].

The results concerning counselling self-efficacy’s significance about attitudes toward tele-mental health indicate a positive and significant association, implying that improvements in counselling self-efficacy are linked to more positive attitudes toward tele-mental health. This finding aligns with prior studies indicating that enhanced confidence among counsellors in their counselling abilities leads to greater engagement with or acceptance of tele-mental health [44,55].

Several prior studies have demonstrated that adequate training in counselling skills enhances counsellors’ confidence levels, enabling them to effectively manage various counselling sessions, including those carried out through tele-mental health platforms. Training programs that focus on both counselling techniques and computer skills are instrumental in fostering counsellor acceptance of tele-mental health services [55].

Previous research has shown that school counsellor’s educational background and confidence levels are strongly linked to their willingness to use online counselling services. This study supports these findings and demonstrates that school counsellors are more likely to engage in online counselling practices when they feel confident and comfortable with the technology [56]. It is worthy to note that training initiatives and educational experiences have been shown to enhance counsellor confidence in applying their counselling skills to online counselling contexts [57]. Through educational training, counsellors can improve their self-efficacy and experiences in counselling and technology use, ultimately leading to greater confidence when conducting online counselling sessions [40].

According to a study conducted by [55], therapists who have extensive experience, are older, and operate in mental health centres are generally more adept at conducting tele-mental health. This is attributed to their heightened levels of self-efficacy, which correlate with greater drive, eagerness to tackle obstacles, and proficiency in resolving complicated issues, even in the face of adversity. Consequently, therapists who exhibit these traits are better equipped to navigate the challenges associated with tele-mental health without succumbing to self-doubt. The results are further supported by the research of [58]. This suggests that counsellors who exhibit experiences and confidence in managing risks during face-to-face counselling sessions are more likely to feel confident when transitioning to tele-mental health services.

Our research has uncovered a promising relationship between individuals’ confidence in using computers and their attitudes towards tele-mental health. This finding is consistent with previous studies conducted by [43] and [59], which highlighted the significance of counsellor’s feeling comfortable and at ease working with computer tools when providing tele-mental health services.

In a more recent study, [56] have identified a strong link between having positive perceptions of technology, possessing advanced computer skills, and using tele-mental health as a part of counselling practice. Their research findings found that counsellors with experience with blended learning, which refers to a combination of online and in-person education, are better prepared to adapt to tele-mental health modalities. This is because they have gained valuable skills and knowledge through their online education, as demonstrated by [40].

As per the findings of [60], resilience appears to be a primary factor in moderating the connection between computer self-efficacy and attitudes toward tele-mental health. Their research suggests that school counsellors with high resilience tend to demonstrate improved computer self-efficacy and a more favourable outlook toward tele-mental health. This correlation can be explained by the fact that resilience has a positive effect on psychological well-being, empowering counsellors to tackle the challenges inherent in tele-mental health practice proficiently [60].

We have extensively explored the connections between counselling self-efficacy, computer self-efficacy, and attitudes toward tele-mental health. Our findings indicate that counselling self-efficacy is vital in mediating the relationship between computer self-efficacy and attitudes toward tele-mental health. To strengthen these relationships, it is imperative to prioritize education and training on tele-mental health, enhance counsellor resilience, and offer skill-building opportunities. Investing in these areas can cultivate stronger connections and encourage favourable perceptions of tele-mental health among counsellors.

### Implication

The findings of this study suggest that integrating counselling techniques and computer skills training into educational programs may be beneficial for preparing counsellor to engage in tele-mental health. The present research does not evaluate specific training programs, but the results highlight potential areas for development in counselling education and professional training. Incorporating computer and tele-mental health skill practice into counselling programs may enhance the confidence and competence of counsellors in utilizing tele-mental health services in the future.

To further enhance the confidence of counsellors in utilising tele-mental health tools, skill-building opportunities tailored to their specific needs could be provided. These could include workshops, seminars, or online courses on enhancing computer literacy and counselling efficacy in virtual environments. By providing such initiatives, we can better equip counsellors with the required skills to deliver effective counselling services in the ever-evolving technological landscape.

Blended learning, a combination of online and in-person education, is an effective strategy to prepare counsellors to use tele-mental health. According to research by [40], counsellors who have undergone blended learning exhibit enhanced readiness to adapt to online counselling practices. Therefore, blended learning should be integrated into degree or master’s degree counselling courses to equip future counsellors with the necessary skills and attitudes to engage effectively in virtual counselling settings. Although this study did not directly assess the impact of blended learning, existing evidence suggests it may be worth considering for integration into counselling degree and master’s programs to support skill development for virtual counselling settings.

To ensure that counsellors are well-equipped to use tele-mental health tools, policymakers and educational institutions can consider incorporating tele-mental health training into counselling curricula. Such training helps practising counsellors keep up with technological advancements and best practices in the field. Policies promoting ongoing professional development in tele-mental health could be implemented to support practising counsellors in enhancing their skills and knowledge.

### Future recommendation

This study has several limitations that should be acknowledged. First, the study only tested one mediator. The use of tele-mental health has been gaining popularity in recent years. To offer more comprehensive insights into its effectiveness, it is recommended that future research should explore additional moderation variables beyond the single mediator tested. Factors such as age, gender, cultural background, clinical experience, and years of experience should be included in future research to better understand the impact on counsellor attitudes toward tele-mental health.

Second, the cross-sectional design prevents causal conclusions about the relationships between computer self-efficacy, counselling self-efficacy, and attitudes toward tele-mental health. To better understand the effectiveness of tele-mental health training, longitudinal studies need to be employed to assess the long-term effects of such training on counsellor attitudes and confidence levels. Implementing such studies can help educators track the effectiveness of training programs and identify areas for improvement. Additionally, although this study focuses on school counsellors, catering to a range of issues encountered by students, future studies could benefit from narrowing the scope to a specific psychological or mental health condition, such as anxiety, depression, or social challenges like bullying and family abuse. Such a focused path would improve understanding of the effectiveness and applicability of online counselling services within the school context.

Third, as the sample was drawn solely from school counsellors in two Malaysian states, the findings should be interpreted with caution and may not be generalized to counsellors nationwide. Comparing attitudes and practices across different cultural contexts is essential in gaining a better understanding of the adoption and acceptance of tele-mental health. This can help identify culturally specific factors that may influence adoption and acceptance and inform the development of culturally sensitive tele-mental health interventions. Therefore, drawing more school counsellors from multiple regions and diverse cultural contexts will allow for comparisons that may identify culturally specific factors influencing tele-mental health adoption.

Finally, this study did not address ethical and legal considerations in tele-mental health practice. Confidentiality, informed consent, and boundary management are some ethical dilemmas encountered in virtual counselling settings that could be explored in future research. Strategies for navigating these challenges can inform professional guidelines and training protocols. Research on ethical dilemmas encountered in virtual counselling settings can also help professionals involved in tele-mental health practice to make informed decisions and ensure that their practices are ethical and legal.

## Conclusion

This study examined the relationship between computer self-efficacy, counselling self-efficacy, and attitudes toward tele-mental health among Malaysian school counsellors. Based on the results obtained, counseling self-efficacy has a partial mediation effect. The study finds that those counsellors who possess a high level of self-confidence in their counselling skills are more inclined to utilize tele-mental health services, and this association is reinforced by counselling self-efficacy. Furthermore, the study emphasizes the significance of counselling and computer self-efficacy in promoting the adoption of tele-mental health practices. Adopting and implementing technology and investing in counsellor development can enhance mental health care access and quality. These insights may inform the design of training programs that simultaneously develop technological competence and counselling confidence by addressing both skill domains, professional development initiatives could support integrating tele-mental health into school counselling practice in Malaysia. Future research employing longitudinal or experimental designs merits further investigation to assess causal relationships and the long-term effects of such training.

## Supporting information

S1 File PLOSOne_Human_Subjects_Research_Checklist-ZaidaNor. (DOCX)

S2 File Supplementary information (anonymized). (DOCX)
