# Supplementary material for: Counselling self-efficacy as a mediator between computer self-efficacy and attitudes toward tele-mental health among school counsellors in Malaysia
Source: PLoS One. 2025 Nov 6;20(11):e0335955. doi: 10.1371/journal.pone.0335955 (PMC12591479; doi:10.1371/journal.pone.0335955)
Supplement: S2 File — (DOCX) [file pone.0335955.s002.docx]

**Correlation**

| **Descriptive Statistics** | | | |
| --- | --- | --- | --- |
|  | Mean | Std. Deviation | N |
| Com01 | 3.4970 | .61791 | 348 |
| Couns02 | 4.0984 | .62177 | 348 |
| Attitu03 | 3.2078 | .54235 | 348 |

| **Correlations** | | | | |
| --- | --- | --- | --- | --- |
|  | | Com01 | Couns02 | Attitu03 |
| Com01 | Pearson Correlation | 1 | .113^*^ | .174^**^ |
|  | Sig. (2-tailed) |  | .036 | .001 |
|  | N | 348 | 348 | 348 |
| Couns02 | Pearson Correlation | .113^*^ | 1 | .319^**^ |
|  | Sig. (2-tailed) | .036 |  | .000 |
|  | N | 348 | 348 | 348 |
| Attitu03 | Pearson Correlation | .174^**^ | .319^**^ | 1 |
|  | Sig. (2-tailed) | .001 | .000 |  |
|  | N | 348 | 348 | 348 |
| *. Correlation is significant at the 0.05 level (2-tailed). | | | | |
| **. Correlation is significant at the 0.01 level (2-tailed). | | | | |

**Mediation effect**

Run MATRIX procedure:

***************** PROCESS Procedure for SPSS Version 4.2 *****************

(writer anonymized)

**************************************************************************

Model : 4

Y : Attitu03

X : Com01

M : Couns02

Sample

Size: 348

**************************************************************************

OUTCOME VARIABLE:

Couns02

Model Summary

R R-sq MSE F df1 df2 p

.1125 .0127 .3828 4.4390 1.0000 346.0000 .0358

Model

coeff se t p LLCI ULCI

constant 3.7023 .1909 19.3968 .0000 3.3269 4.0777

Com01 .1133 .0538 2.1069 .0358 .0075 .2190

Standardized coefficients

coeff

Com01 .1125

**************************************************************************

OUTCOME VARIABLE:

Attitu03

Model Summary

R R-sq MSE F df1 df2 p

.3482 .1212 .2600 23.7931 2.0000 345.0000 .0000

Model

coeff se t p LLCI ULCI

constant 1.6936 .2273 7.4521 .0000 1.2466 2.1406

Com01 .1227 .0446 2.7530 .0062 .0350 .2104

Couns02 .2647 .0443 5.9751 .0000 .1776 .3519

Standardized coefficients

coeff

Com01 .1398

Couns02 .3035

************************** TOTAL EFFECT MODEL ****************************

OUTCOME VARIABLE:

Attitu03

Model Summary

R R-sq MSE F df1 df2 p

.1740 .0303 .2861 10.8015 1.0000 346.0000 .0011

Model

coeff se t p LLCI ULCI

constant 2.6737 .1650 16.2041 .0000 2.3492 2.9983

Com01 .1527 .0465 3.2866 .0011 .0613 .2441

Standardized coefficients

coeff

Com01 .1740

************** TOTAL, DIRECT, AND INDIRECT EFFECTS OF X ON Y **************

Total effect of X on Y

Effect se t p LLCI ULCI c_cs

.1527 .0465 3.2866 .0011 .0613 .2441 .1740

Direct effect of X on Y

Effect se t p LLCI ULCI c'_cs

.1227 .0446 2.7530 .0062 .0350 .2104 .1398

Indirect effect(s) of X on Y:

Effect BootSE BootLLCI BootULCI

Couns02 .0300 .0149 .0026 .0619

Completely standardized indirect effect(s) of X on Y:

Effect BootSE BootLLCI BootULCI

Couns02 .0342 .0163 .0030 .0674

*********************** ANALYSIS NOTES AND ERRORS ************************

Level of confidence for all confidence intervals in output:

95.0000

Number of bootstrap samples for percentile bootstrap confidence intervals:

5000

------ END MATRIX -----
